# Improving the Accuracy of Ensemble Machine Learning Classification Models Using a Novel Bit-Fusion Algorithm for Healthcare AI Systems

**DOI:** 10.3389/fpubh.2022.858282

**Published:** 2022-05-04

**Authors:** Sashikala Mishra, Kailash Shaw, Debahuti Mishra, Shruti Patil, Ketan Kotecha, Satish Kumar, Simi Bajaj

**Affiliations:** ^1^Symbiosis Institute of Technology, Symbiosis International University, Pune, India; ^2^Department of Computer Science and Engineering, Siksha O Anusandhan Deemed to be University, Bhubaneshwar, India; ^3^Symbiosis Centre for Applied Artificial Intelligence (SCAAI), Symbiosis Institute of Technology, Symbiosis International (Deemed University), Pune, India; ^4^School of Computer Data and Mathematical Sciences, University of Western Sydney, Sydney, NSW, Australia

**Keywords:** bit-fusion ensemble algorithm, classifier fusion, k-nearest neighbor, Multi-Layer Perceptron, Naïve Bayesian classifier, support vector machine

## Abstract

Healthcare AI systems exclusively employ classification models for disease detection. However, with the recent research advances into this arena, it has been observed that single classification models have achieved limited accuracy in some cases. Employing fusion of multiple classifiers outputs into a single classification framework has been instrumental in achieving greater accuracy and performing automated big data analysis. The article proposes a bit fusion ensemble algorithm that minimizes the classification error rate and has been tested on various datasets. Five diversified base classifiers k- nearest neighbor (KNN), Support Vector Machine (SVM), Multi-Layer Perceptron (MLP), Decision Tree (D.T.), and Naïve Bayesian Classifier (N.B.), are used in the implementation model. Bit fusion algorithm works on the individual input from the classifiers. Decision vectors of the base classifier are weighted transformed into binary bits by comparing with high-reliability threshold parameters. The output of each base classifier is considered as soft class vectors (CV). These vectors are weighted, transformed and compared with a high threshold value of initialized δ = 0.9 for reliability. Binary patterns are extracted, and the model is trained and tested again. The standard fusion approach and proposed bit fusion algorithm have been compared by average error rate. The error rate of the Bit-fusion algorithm has been observed with the values 5.97, 12.6, 4.64, 0, 0, 27.28 for Leukemia, Breast cancer, Lung Cancer, Hepatitis, Lymphoma, Embryonal Tumors, respectively. The model is trained and tested over datasets from UCI, UEA, and UCR repositories as well which also have shown reduction in the error rates.

## Introduction

Classification plays a vital role in identifying the pattern and accuracy in pattern recognition (1, 2). Classifier accuracy depends on dimension and type of data set. Single classification techniques are not capable enough to handle huge data. Sometimes the accuracy level changes according to the number of classifiers employed. The single classifier is not competent enough to always get the targeted accuracy level. To overcome this problem, fusion algorithms have been introduced. It takes the output from the multiple classification algorithms and determines the class level's accuracy. Machine learning-based classification models have improved accuracy by combining the results of multiple ML algorithms. Such an ensemble approach has been explored widely in various application domains such as computer vision, natural language processing and pattern recognition. Ensemble or fusion methods consider the output of each classifier as input. It considers the class level accuracy collected from all classifiers rather than the whole dataset. The model has to run all classification algorithms. It takes more time but efficacy increases. However, only ensembling the results obtained by the ML algorithms is not always beneficial. During the pandemic, it was realized that segregating patterns to detect the type of patient was very challenging.

Different fusion approaches (3–7) have failed to provide better accuracy, focusing only on ensemble algorithms. In this article, the authors have proposed a bit fusion method wherein the model trained itself to merge soft class labels (1). It uses its strategies to update weight, bias, and other parameters. Last decade we witnessed many such fusion classification techniques built and tested over other datasets. The proposed bit fusion model considers the input as soft class level and finds the accuracy.

This article describes the proposed model's complete theoretical and realistic aspects to establish the incorporation of fusion strategy with the established classifier. Literature review introduces the importance of base classifiers and the background work done. Methodological foundations, the structural and functional concept of the bit-fusion classifier are presented in proposed framework. Experimental assessment and simulation results discusses experimental evaluation with data set description and parameter discussion, and finally, conclusion deals with the conclusion and outlook of the work.

## Literature Review

**Figure 1:**
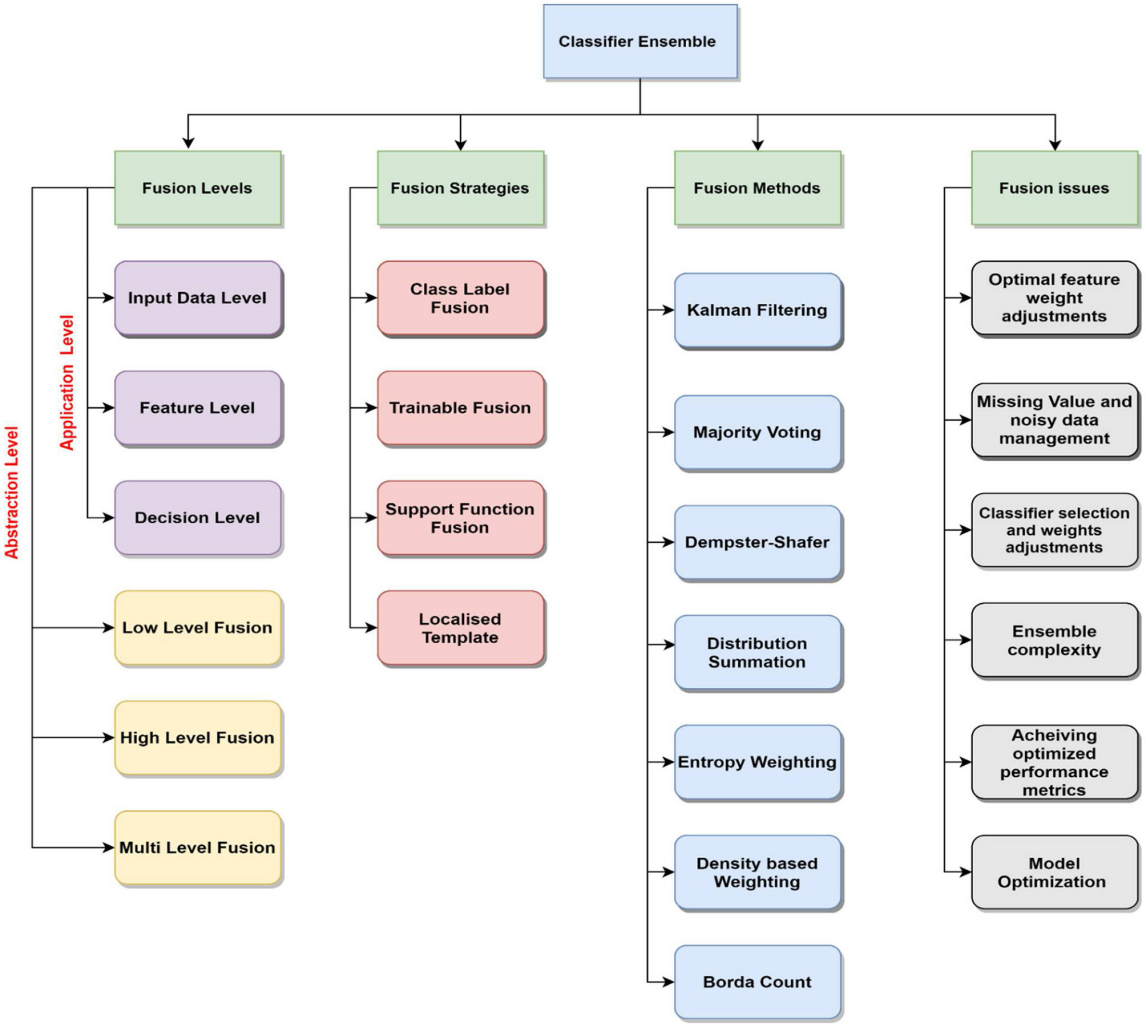
Overview of existing classifier ensemble domain.

In the last decade, various researchers have proposed combining the results of multiple classifiers to achieve better model performance in diverse application domains. This area of research has witnessed the development of miscellaneous model output combination strategies such as Bagging, Boosting, majority voting, Dempster-Shafer, etc. This has improved the accuracy percentage but still has more scope for improving the logical fusion framework. Figure 1 showcases a taxonomy of classifier ensemble methods that encompass the fusion methods, levels, strategies, and issues.

Hazem and Bakry (4) has proposed an algorithm for efficient face detection using an amalgamation of multiple classifiers and fusion of input data. The classifiers are designed to analyze the pattern between the input image matrix and the weights matrix of the neural networks. The normalization of the weights was done in the offline mode, which improved face detection accuracy.

Zhang and Yang (5) proposed a hybrid ensemble model and a multi-objective genetic algorithm. They optimized the classifier feature selection using a genetic algorithm and tested on three different benchmarked datasets, improving bagging and boosting ensemble methods. Kittler (6) proposed a solution for fusing the classifiers that utilize different patterns representations, and all of them are considered for doing joint decision making. They proposed combining the multiple patterns that are an output of the individual classifiers and comparing the unique measurement vectors for compound classification.

Enriquez et al. (8) have measured the performance of different fusion approaches such as voting, Bayesian merging, bagging, stacking, feature sub-spacing, and cascading for part of speech using a complete collection of writings in five languages. Both stacking and cascading have shown good accuracy in all cases. Shah and Jivani (9) have explained the algorithms like Decision Tree (D.T.), Bayesian Network (B.N.), and k-nearest neighbor (KNN) algorithms for the prediction of breast cancer. The results are compared for the classification algorithms with the various parameters like relative absolute error, time taken by the algorithm, kappa statistic, and root relative squared error. The authors found that probability-based Bayes classification has more accuracy and less time complexity.

Opitz and Maclin (10) presented the results of bagging and boosting as an ensemble method for neural networks and decision trees. The results showed that a boosting ensemble could perform better than bagging in a single classifier. Ali Bagheri et al. (11) have evaluated the performance of different classifiers, which has been trained with various feature sets of images. The accuracy of fused classifiers increases on individual classifiers. Dempster Shafer fusion was used for the fusion approach to establish the accuracy by the authors.

The soft class label is the class predicted by the intermediate classifier. Sohn and Lee (12) have used data fusion, ensemble, and clustering to increase the performance of classification algorithms for road traffic accidents in Korea. The authors have used neural networks and decision trees to find the classification model for road traffic accidents, but the accuracy of individual classifiers was ranges between 72 and 79%. To enhance the competency level of the model data fusion algorithm has been used by the authors.

Recently few more ensemble based learning model has been proposed in various domains such as health care (13), medical data analysis (14), medical record linkage (15), feature selection model (16), and health care recommendation for diabetes patients (17). Saxena et al. (13) have applied various classifier logistic regression, decision tree, random forest, KNN, support vector machine (SVM), and Naïve Bayes method on health care dataset, finally fused the results with majority voting for prediction of human health changes. Namamula and Chaytor (14) integrated “Edge Detection Instance preference (EDIP)” and “Extreme Gradient Boosting (XGboost)” fused with voting techniques to analysis large scale medical data. Vo et al. proposed a record linkage (15) for identifying unique patient across multiple care through fusion of three classifier SVM, logistic regression, standard feed forward neural network over synthetic dataset. Nagrajan et al. (16) deals with feature extraction techniques using bio-inspired algorithm and classification using SVM random forest, Naïve Bayes, and decision tree. The authors adopted a fusion approach to combine the output of Learner (17) regression classifier, Naïve Bayes, Random forest, KNN, Decision tree, SVM for prediction of diabetic patients.

Learning outcome of survey is, classifier accuracy and reliability can change with respect to data to data, parameter and training and testing environment. Hence, to increase the reliability and accuracy of model ensemble techniques are proposed to fuse the result of base classifier and get better accuracy. The existing fusion algorithms uses the variety of Classification model as per dataset whereas the proposed Bit-fusion approach analyze the data of a particular feature for the enhancement of the performance of the model. Feature wise fusion is very much applicable to the variety of dataset.

## Proposed Framework

**Figure 2:**
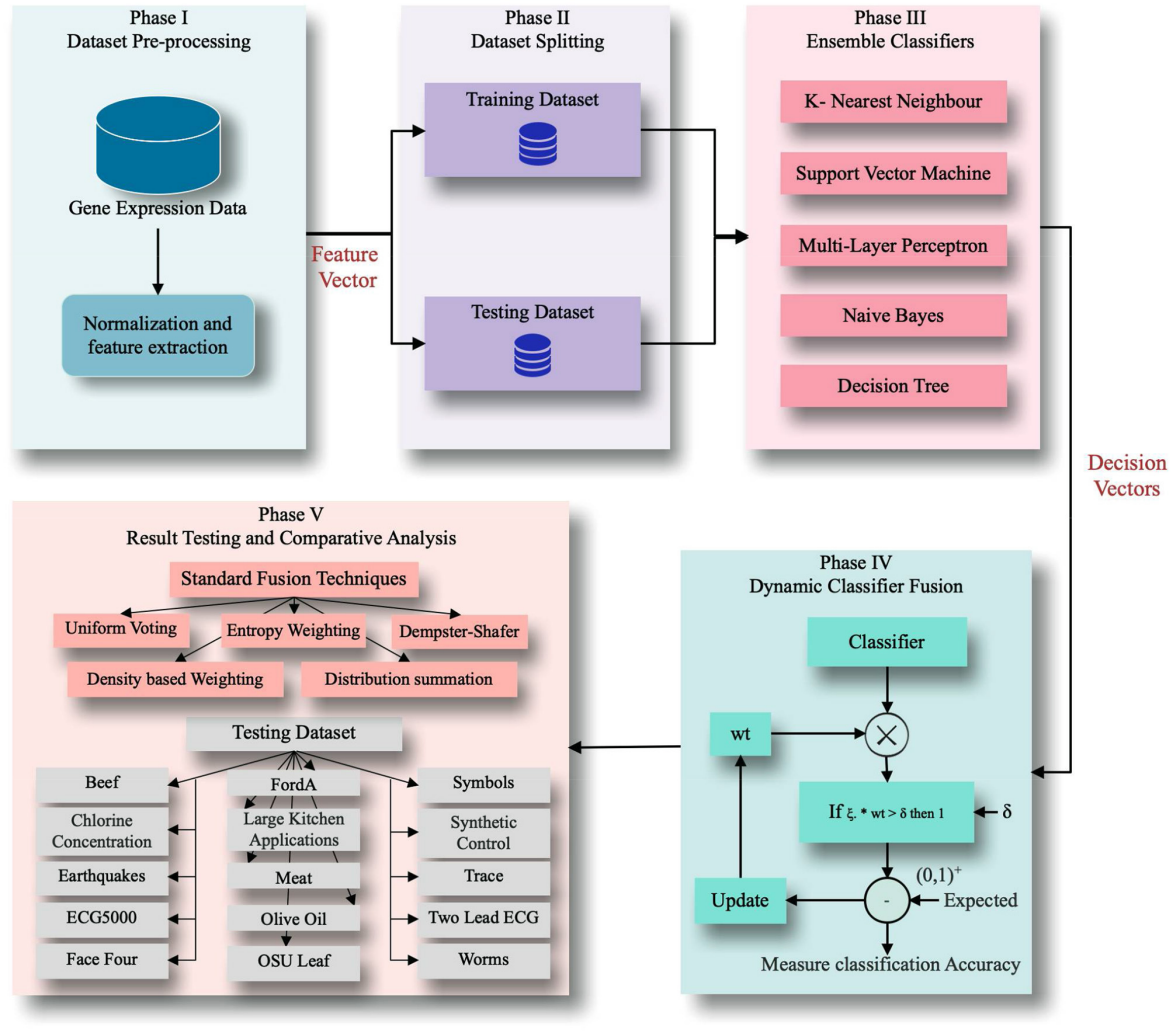
Proposed framework.

Figure 2 shows the framework of the proposed application. It accepts the decision of various classifiers, trains with those decisions and tests the model with weighted transformation. The result is compared with the threshold value for binary equivalence, which allows training the model by updating the weights matrix. Results are tested and compared on a dataset downloaded from KDD, UCI repository, and state-of-the-art algorithms.

### Significance of Base Classifier

The proposed model employs five different base classifiers to outline the application of bit-fusion classifier methodology for enhancing the framework's efficiency. The model takes the input from the five base classifiers, which have been implemented in the model. The importance of the base classifiers has been discussed in this section.

A decision tree is fundamentally valuable for indecisive situations. The number of decision trees constructed for the dataset and rules is framed based on the condition. The path to be selected provides the lowest cost (18, 19) within the uncertain situation (20). K-Nearest Neighbor (KNN) is a learning algorithm, but it takes more time for classifying the dataset. It memorizes the details rather than learn through the training data (21). KNN used majority voting rather than training data. It uses the distance learning algorithm to find the closest neighbor. Multi-Layer Perceptron (MLP) is a learning process that easily handles complex data. It uses various layers to train the system during the training phase. The various function is used to predict the class level by tuning the parameters weight and bias to enhance the algorithm's performance (22). Each input is considered a neuron, and the neurons are multiplied by the weight. The activation function is used to predict the class level (23–26). A Naive Bayesian algorithm uses the conditional probability methodology to predict the class level. It's based on the statistical methodology and predicts the class level as per the target value. The value is predicted within 0–1 (27–29). SVM is a supervised learning technique that uses different types of kernel functions to handle multi-class problems such as linear, polynomial, RBF, and sigmoid (30–34). SVM has been widely used to solve pattern recognition problems due to its effectiveness in using those kernels to handle multi-class issues. It can also obtain an optimized margin to separate the classes.

### Bit-Fusion Algorithm Description

This section presents a bit fusion algorithm with the theoretical layout and the working principle. The projected work of the proposed algorithm is discussed in detail with the various parameters.

#### Bit-Fused Ensemble Framework Algorithm

The bit fusion algorithm is applied to the trained classifier. The fusion algorithm considers the output of the classification algorithm to target maximum conceivable accuracy by reducing the execution time. For example, let *Classifier* = {*C*_1_, *C*_2_, .., *C*_*k*_} is the set of *k* number of classifiers, *X* = {*x*_1_, *x*_2_, …, *x*_*n*_} be the input features of the dataset *x*_*i*_ ∈ *R*^*n*^ of *n* instances; where each features can have *m* conditions and set of class labels ω = {ω_1_, ω_2_, …, ω_*p*_}. Individual classification algorithm are trained and tested on input feature *X*. Each classifier reads an input *x*_*i*_ and predict class category ω. i.e., *C*_*i*_(*x*) ∈ ω, for *i* = 1 … *k*. For all the *k* classifier we will have *p* dimension vector supporting the class labels as given in (1).


(1) $$\begin{array}{l} C_{i}\left(X\right)={\left[c_{i,1}\left(x\right),\ldots ,c_{i,p}\left(x\right)\;\right]}^{T} \end{array}$$

Where, *c*_*ij*_(*x*) ∈ [0, 1] for *i* = 1 … *k, j* = 1 … *p* usually provides the soft class labels for the classification algorithm. Thus *c*_*ij*_ denotes the degree of support given by the individual classifier *c*_*i*_ to the hypothesis that *x* belongs to ω_*j*_. Merging classifiers methodology signifies finding a class category for the input *x* based on the *k* number of classifiers outcomes *C*_1_(*X*), *C*_2_(*X*), …, *C*_*k*_(*X*), the output is observed as a vector with final degree as support to the classes as soft class label for *x*, denoted in (2).


(2) $$\begin{array}{l} C\left(X\right)={\left[\mu_{1}\left(x\right),\ldots ,\mu_{p}\left(x\right)\;\right]}^{T} \end{array}$$

The maximum membership rule is applied to get the crisp class label *x* of a data set. Assign *x* to class ω_*s*_ if,


(3) $$\begin{array}{l} \mu_{s}\left(x\right)\geq \mu_{t}\left(x\right),\;\forall \;t=1,..,p \end{array}$$

There are two strategies of classifier combination such as classifier selection (2, 35) and classifier fusion (36–39). The belief in classifier selection is that each classifier has expertise in some local area of the feature space. When a feature vector *x* ∈ ℜ^*n*^ is submitted for classification, the classifier responsible for the vicinity of x is given the highest authority to label ω. Classifier fusion assumes that all classifiers are equally exposed to the whole feature space and the decisions of all of C are taken into account for any x. The classifier resulting from bit- fusion is a classifier fusion technique which is in the remainder of this article. Algorithm 1 is overview of the proposed algorithm.

**Algorithm 1:** Bit-fusion ensemble algorithm.

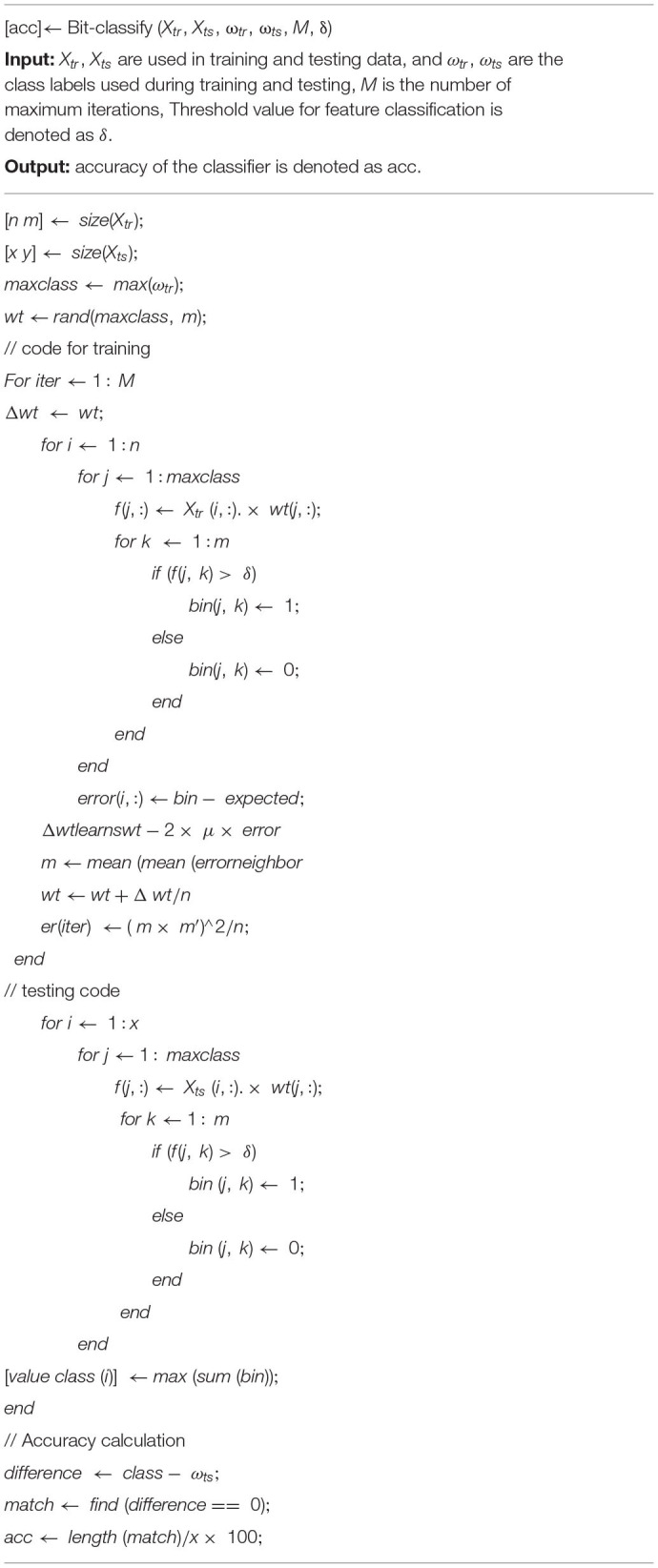

### Algorithm Steps

The framework of the bit-fusion ensemble process is given in Figure 1. The proposed model mechanism is described in three segments as below:

Phase 1: Min-Max Normalization and feature extraction: Min-Max normalization (4) is used to normalize the input feature X∈ *R*^*n*^. Min-max normalization is the traditional way to transform input features into the scale of [0, 1]. The minimum value of the feature is transferred to 0, whereas the maximum value is converted to 1, rest of the values are transformed between 0 and 1.


(4) $$\begin{array}{l} x_{ij}=\frac{x_{ij}-min\left(X_{j}\right)}{max\left(X_{j}\right)-min\left(X_{j}\right)} \end{array}$$

where *x*_*ij*_ ∈ *X*.

Principal component analysis (PCA) is used for feature extraction. It is done using three steps: (1) Covariance matrix (*Z*) using Equation (5), (2) Compute eigenvalue and eigenvector *U* of *Z* using Equation (7) and (3) project the row data into k-dimensional subspace using Equation (8).


(5) $$\begin{array}{l} Z=\;\frac{1}{n}\sum_{i}^{n}x_{i}x_{i}^{T},\;Z\in R^{m\;\times \;m} \end{array}$$

(6) $$\begin{array}{l} u^{T}Z=\;\lambda u \end{array}$$

(7) $$\begin{array}{lll} U & = & \left\{\begin{array}{ccc} | & | & |\\ u_{1} & u_{2} & .\\ | & | & | \end{array}\;\begin{array}{ccc} | & \;| & |\\ . & \;. & u_{m}\\ | & \;| & | \end{array}\right\},\;u_{i}\in R^{m} \end{array}$$

(8) $$\begin{array}{lll} x_{new}^{i} & = & \;\left[u_{i}^{T}x_{i}\;u_{2}^{T}x_{i}\;\ldots \;u_{k}^{T}x_{i}\;\right]\in R^{k} \end{array}$$

Where row data was m dimensions, and new features are of *k* dimension.

Phase 2: Classifier Building: Considering the literature review, all ensemble technique has fixed few base classifiers and applied fusion using ensemble techniques. Similarly, in the proposed experiment, we have employed *l* = 5 classifiers N.B., D.T., SVM, k-NN and MLP as base classifier. For an dataset of *n* features and *l* classifiers we get soft class label output matrix as ξ of dimension *l* × *n* as shown in (9).


(9) $$\begin{array}{l} \xi =\;\left[\begin{array}{ccc} C_{11} & \cdots & C_{n1}\\ \vdots & \ddots & \vdots \\ C_{1l} & \cdots & C_{nl} \end{array}\right] \end{array}$$

Where *C*_*ij*_ ∈ ω is the class level predicted by *the j*^th^ classifier for the *i*^th^ feature.

**Figure 3:**
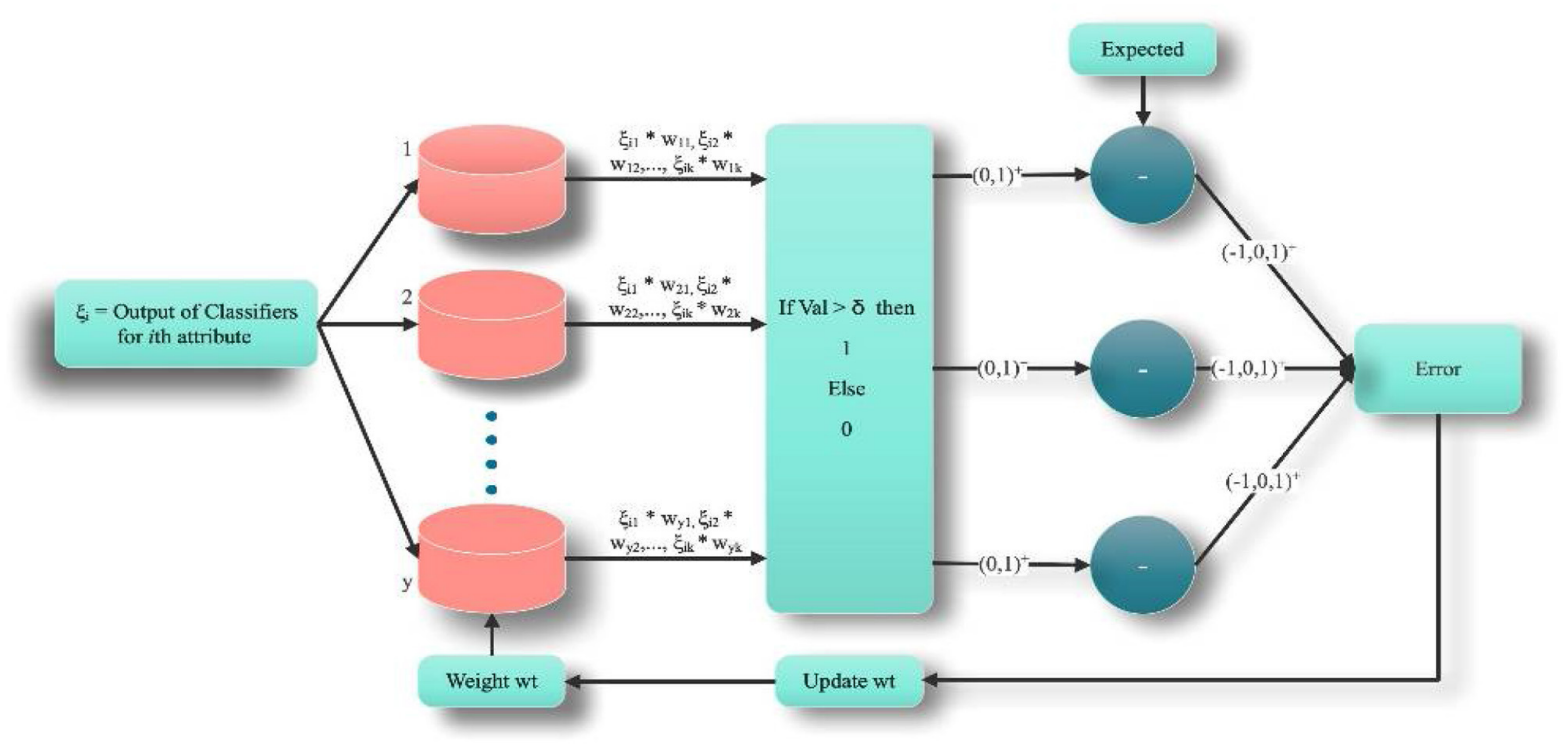
Working procedure for bit-fused classifier.


Phase 3: Training of bit-fusion classifier: The bit fusion classification algorithm is used to categorize every value. In Figure 3, ξ is treated as an input to the fusion method, and for 100 iterations, it's trained for the given feature input. In every epoch, all occurrences of the data set ξ contribute to the model's training. Let ξ_*i*_ Represents each classification result from an individual classifier by considering the *i*^th^ feature. A random value between [−0.5, 0.5] is selected to tune the Weight wt. Dimension of *wt* is set to |ω| × *l*, where l denotes the number of classifiers. Each row in *wt*_*ij*_ is tuned for ξ_*gc*_. Initially, the dot product of the *f* (ξ_*i*_W*t*) is evaluated using Equation (10).

(10) $$\begin{array}{l} \text{f}\left(\xi_{i},\text{wt}\right)=\xi_{i}.\times \text{wt} \end{array}$$

Now, binary output *B* is evaluated by comparing *f* (.) with high threshold parameter δ. In the proposed algorithm, we have initialized δ = 0.9. For better training, the value is considered near to 1. For all values of *f* (ξ_*i*_W*t*) > δ is set to as 1 otherwise 0. Let B (*f* (ξ_*i*_, *wt*), δ) be the function that relates the value of *f* (ξ_*i*_W*t*) with δ and set it to binary value {0, 1}.


(11) $$B\left(f\left(\xi_{i},wt\right),\;\delta \right)=\{\begin{array}{c} 1\\ 0 \end{array}\;\begin{array}{c} f\;(\xi_{i},\;wt)\;>\;\delta \\ Otherwise \end{array}$$

B(*f*(ξ_*i*_, *wt*), δ) is compared with the expected output to evaluate model training error. Training error can be assessed using Equation (12).


(12) $$\begin{array}{l} E_{iter,\;i}=B\left(f\left(\xi_{i},\;wt\right),\;\delta \right)-\omega_{i}\;\forall i\epsilon 1,\ldots ,n \end{array}$$

Model learning is done by updating *wt* using Equation (14).


(13) $$\begin{array}{l} \Delta wt=\;\eta \;\times \;wt\;\times \mu \;\times Error \end{array}$$

(14) $$\begin{array}{l} wt=wt+\frac{\Delta wt}{n} \end{array}$$

Where η and μ are the *coefficients of learning* and *accelerator* adjusted and initialized to 0.71 and 0.00001.

Equations (10–13) is repeated for maximum Iteration. We set a *maximum number of iterations* as 100. The Mean square error for *j*^th^ epoch is evaluated using (15) and stored in φ(j).


(15) $$\begin{array}{l} \varphi \;\left(j\right)={\left(E/n\times \;E/n\right)}^{2/n} \end{array}$$

**Figure 4:**
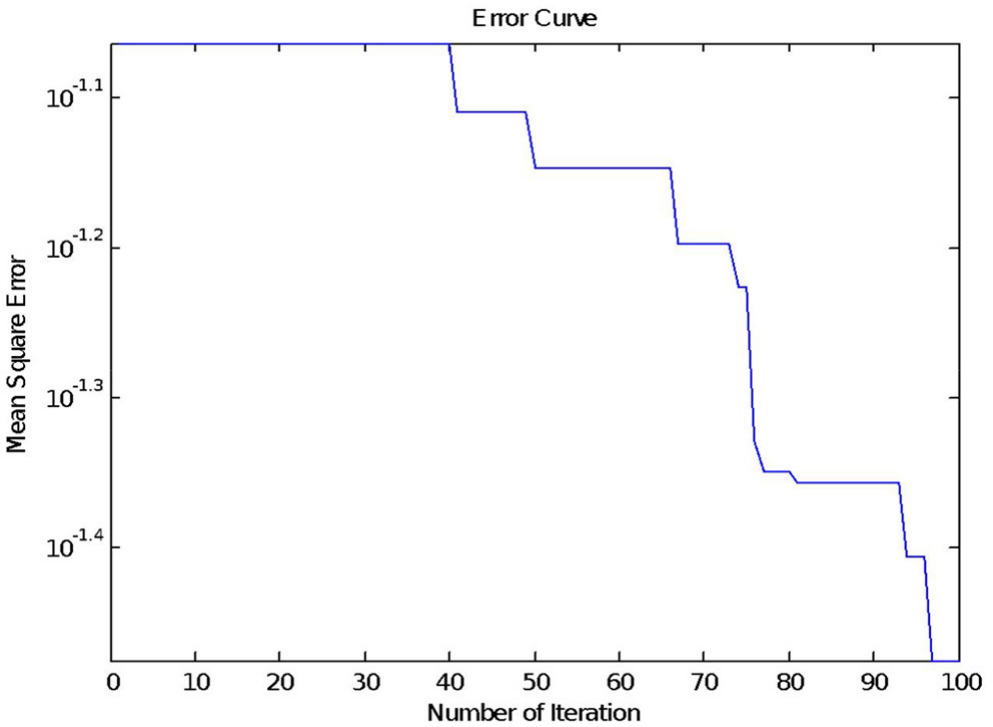
Mean square error on Leukemia Dataset.

Figure 4 shows the mean square error with iterations, where the x-axis represents several iterations and the y-axis represents the *mean square error*, φ.

After the model training step, classifier performance is tested on testing data. The classifier generated output ξ′ are analyzed by the bit-fusion classifier. F(ξ, *wt*)′ is calculated using (10). Binary sequence B(*f* (ξ, *wt*), δ) ′ is generated by comparing *f* (ξ, *wt*) with δ. Then final prediction *p*(ξ) is done by using (16).


(16) $$\begin{array}{l} p\left(\xi^{\prime }\right)={\text{max}}_{i\epsilon T}\sum_{j=1}^{k}B_{ij}^{\prime } \end{array}$$

## Experimental Assessment and Simulation Results

### Details of Datasets

**Figure 5:**
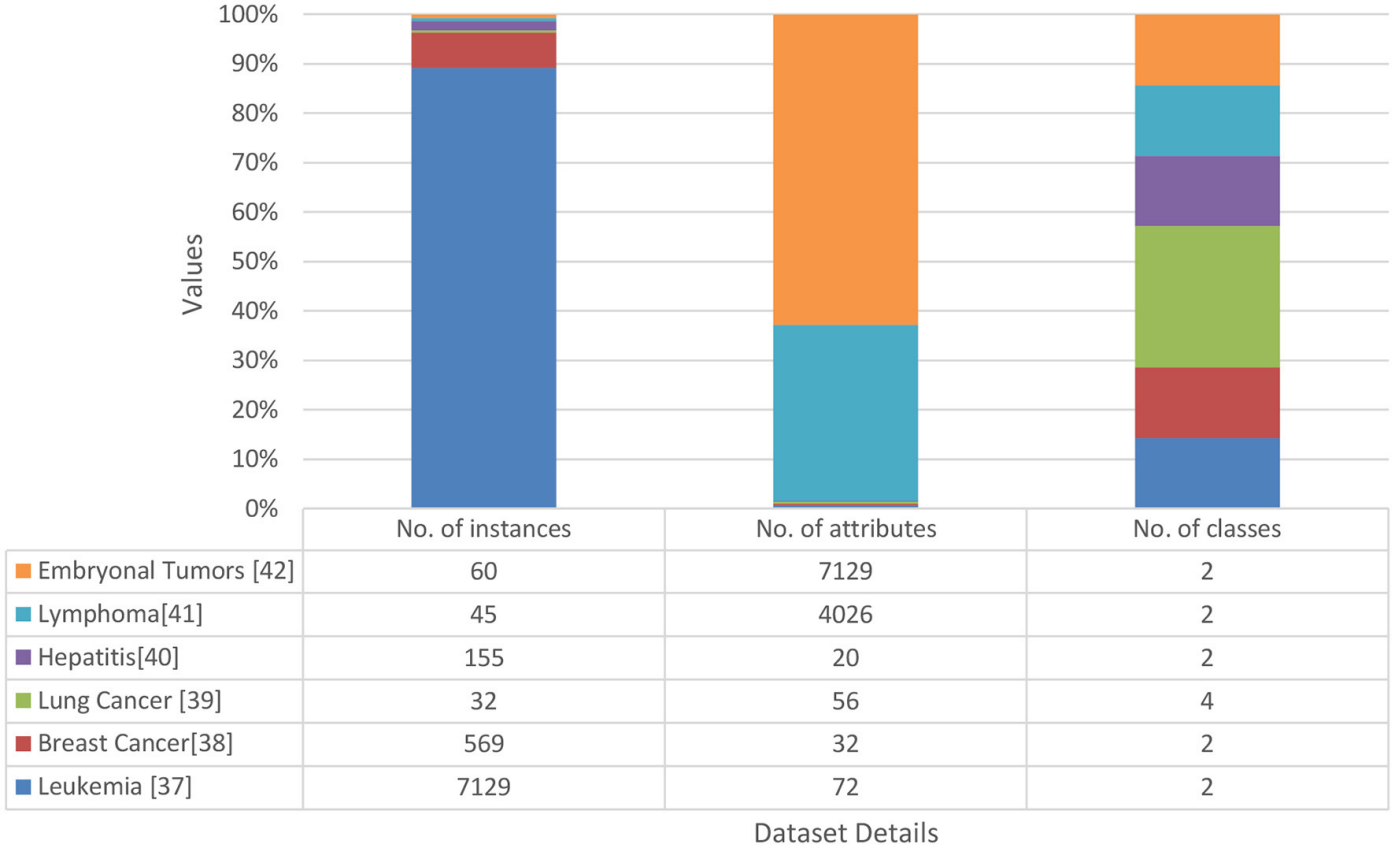
Data sets used for experimental evaluation.

**Figure 6:**
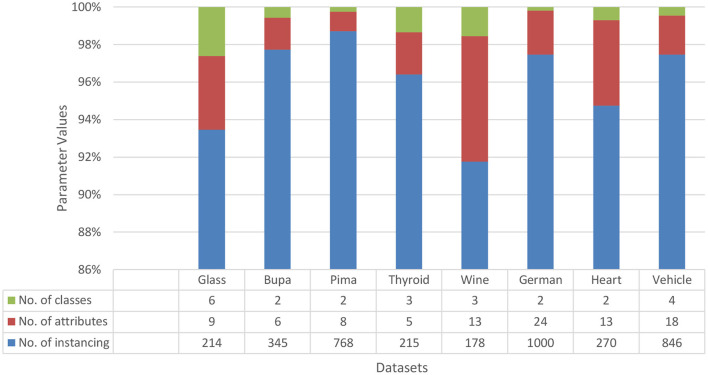
Data sets for comparison with D.T. and VWDT.

**Figure 7:**
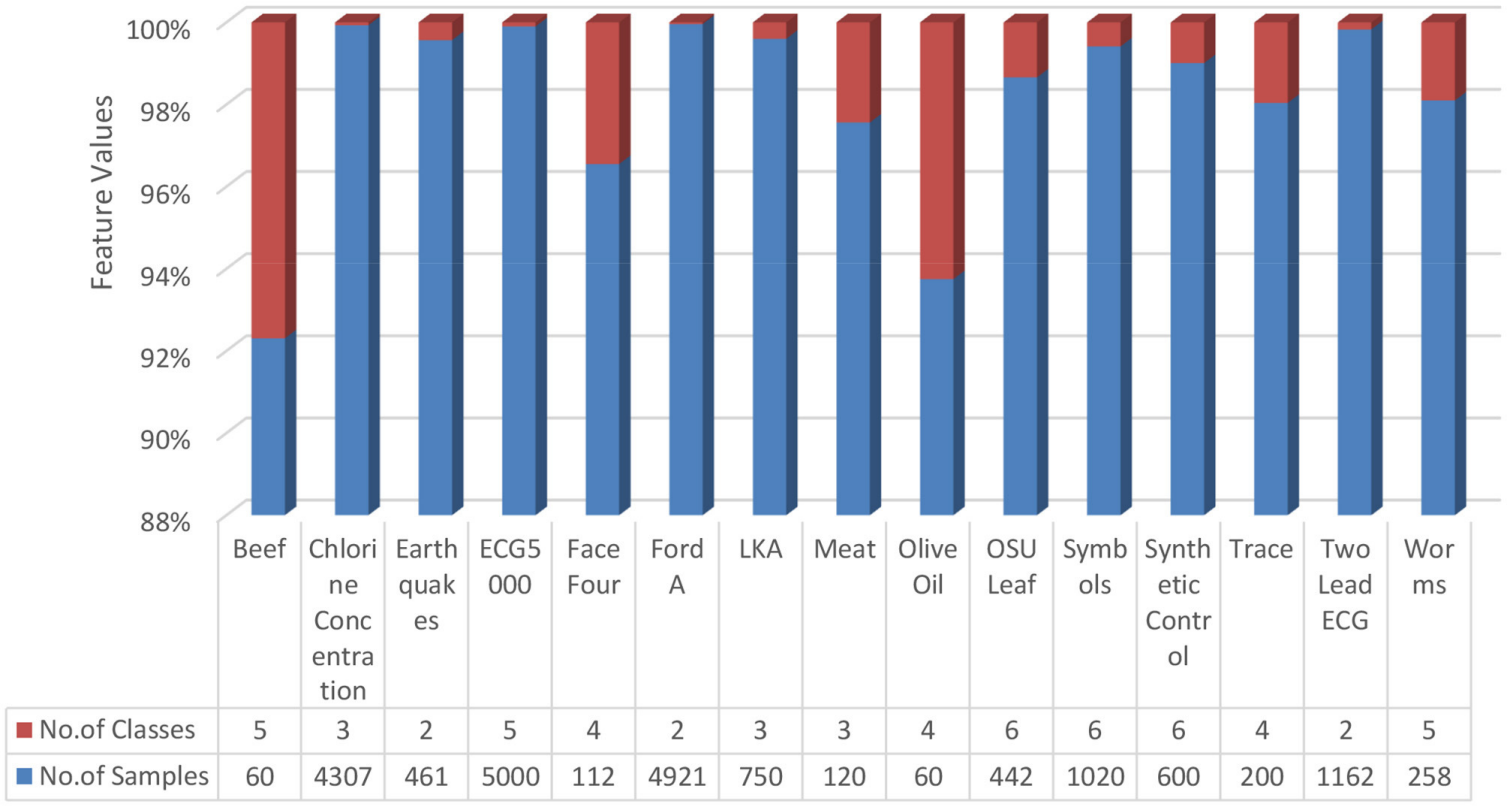
Dataset from UEA and UCR (48).

Fourteen data sets were collected from the standard repository database (https://archive.ics.uci.edu/ml/datasets.php) to analyze and establish the accuracy of the proposed model. Dataset selected from the repository does not have any missing values features. Normalization has been used on the dataset to improve accuracy and avoid the model's biasing (40–47). The basic details of dataset dimension and class levels, attributes and instances are provided in Figures 5, 6. To establish the correctness of our model, testing has been done on another 15 datasets collected from UEA and UCR (48). Figure 7 provides the details about the testing dataset. The dataset does not have the missing value; it uses the standard scaler with zero mean and unit S.D.

### Parameter Discussion

The proposed methodology and five classifiers have been applied on 29 benchmarked datasets, as shown in Figures 5–7. The average error rate has been calculated by employing a 10-fold cross-validation test shown in Tables 1, 2. The result visualization represents an accuracy improvement on all the datasets. The entire implementation pipeline was developed and tested in Matlab R2010a.

**Table 1:** Parameter summary.

**Sl. No**	**Name of dataset**	**MLP**		**SVM**		**BFC**	
		**η **	**μ **	**C**	**γ**	**η**	**μ**
1	Leukemia	0.4	0.5	2^3^	2^−15^	0.5	0.2
2	Breast cancer	0.6	0.2	2^−1^	2^−6^	0.2	0.1
3	Lung cancer	0.5	0.5	2^3^	2^−10^	0.3	0.5
4	Hepatitis	0.2	0.5	2^3^	2^−15^	0.4	0.4
5	Lymphoma	0.3	0.3	2^−5^	2^−10^	0.2	0.3
6	Embryonal tumors	0.4	0.4	2^−1^	2^−3^	0.6	0.2
7	Glass	0.3	0.2	2^1^	2^−8^	0.2	0.2
8	Bupa	0.2	0.5	2^5^	2^−3^	0.1	0.2
9	Pima	0.5	0.2	2^1^	2^−4^	0.3	0.3
10	Thyroid	0.4	0.4	2^1^	2^−6^	0.1	0.2
11	Wine	0.6	0.1	2^−4^	2^−8^	0.5	0.2
12	German	0.4	0.5	2^−1^	2^−2^	0.4	0.3
13	Heart	0.5	0.2	2^3^	2^−4^	0.3	0.1
14	Vehicle	0.2	0.2	2^−3^	2^−3^	0.4	0.1
15	Beef	0.4	0.5	2^2^	2^−4^	0.4	0.2
16	Chlorine concentration	0.6	0.5	2^2^	2^−8^	0.5	0.4
17	Earthquakes	0.4	0.1	2^−3^	2^−3^	0.2	0.4
18	ECG5000	0.3	0.4	2^−2^	2^−2^	0.3	0.2
19	Face four	0.2	0.2	2^2^	2^−6^	0.1	0.3
20	Ford A	0.4	0.5	2^−1^	2^−4^	0.4	0.3
21	Large kitchen appliances	0.2	0.3	2^3^	2^−5^	0.2	0.1
22	Meat	0.4	0.4	2^3^	2^−8^	0.4	0.4
23	Olive oil	0.6	0.2	2^−4^	2^−1^	0.3	0.1
24	OSU leaf	0.4	0.2	2^−2^	2^−4^	0.5	0.5
25	Symbols	0.5	0.2	2^2^	2^−1^	0.2	0.2
26	Synthetic control	0.2	0.5	2^−2^	2^−3^	0.5	0.3
27	Trace	0.4	0.3	2^4^	2^−1^	0.5	0.2
28	Two lead ECG	0.1	0.3	2^4^	2^−2^	0.2	0.3
29	Worms	0.5	0.4	2^−3^	2^−3^	0.1	0.2

**Table 2:** Performance on the UCI data sets (in %).

**Dataset name**	**Average error rate**					
	**Uniform voting**	**Distribution summation**	**Dempster-shafer**	**Entropy weighting**	**Density-based weighting**	**Proposed algorithm**
Leukemia	15.02	12.54	10.25	14.26	15.02	5.97
Breast cancer	16.3	15.64	16.48	19.44	16.48	12.6
Lung cancer	7.77	12.21	13.53	12.21	12.21	4.64
Hepatitis	5.43	5.43	1.47	7.43	4.06	0
Lymphoma	5.69	7.1	9.32	7.25	4.01	0
Embryonal tumors	28.84	37.84	37.84	33.44	28.84	27.88

Training standard classification algorithms such as MLP, NB, SVM, D.T., KNN and BFC parameter tuning for better results. MLP, SVM, and BFC parameters need to tune properly for better results out of all five classifiers and proposed BFC algorithms. KNN solely depends on value of K neighbors. We use Euclidean distance from the query features to rest of dataset and considered (k = 10) nearest neighbors for voting. N.B. is based on the prior probability of different classes on training data. Details of parameter values are shown in Table 1. The two parameters η and μ, as known as the learning rate and acceleration constant, respectively, the value has been initialized from [0.1, 0.6] to train the MLP algorithm. SVM is a linear classifier that works well on a large data set. It's easier to fit the data as SVM does not depend on native optima. The linear function is used for binary classification. By considering the variety of datasets with multi-class levels, it's difficult to select a proper radial function for data. SVM parameter scales properly to handle the large data set. This article uses the exponential radial function (2, 31–34) in SVM to train dataset. SVM with RBF uses the parameter {C, γ} but it varies as per the data set. The value of C ranges from as per data {2^−5^, 2^−4^, ., ., ., 2^5^} and the value of γ selected for the data set {2^−15^, 2^−14^, …, 2^−1^}. For training and testing, 10-fold cross-validation enhances the accuracy level. The algorithms implemented for all three data sets are presented in the Table 1. The proposed Bit fusion algorithm trains the parameters *acceleration constant* μ and *learning rate* η with the value [0.1, 0.5] [0.1, 0.6] respectively.

### Evaluation of Proposed Bit-Fusion Ensemble Technique With Traditional Fusion Methods

**Figure 8:**
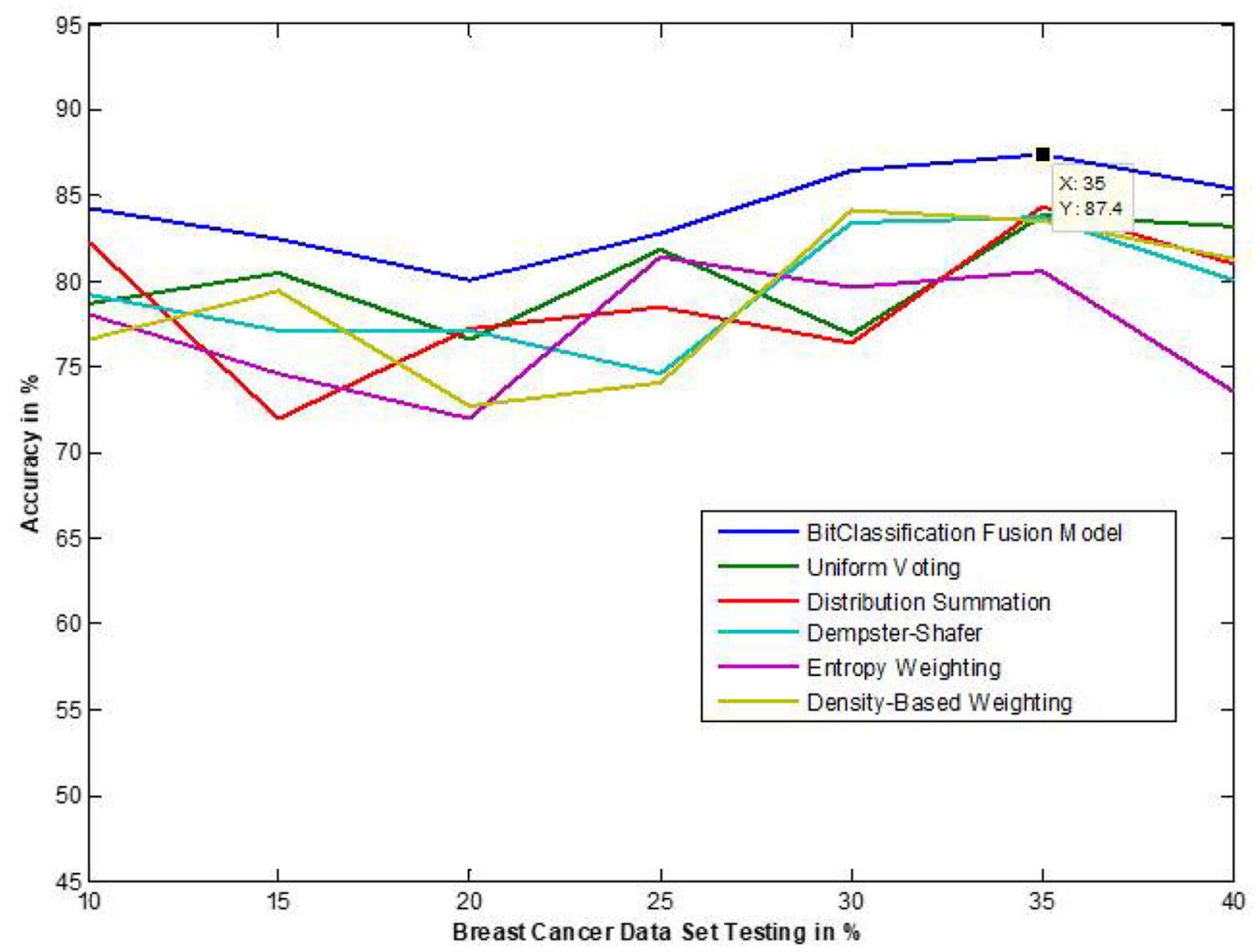
Accuracy performance bit fusion algorithm for Breast cancer with respect to other standard fusion techniques.

**Figure 9:**
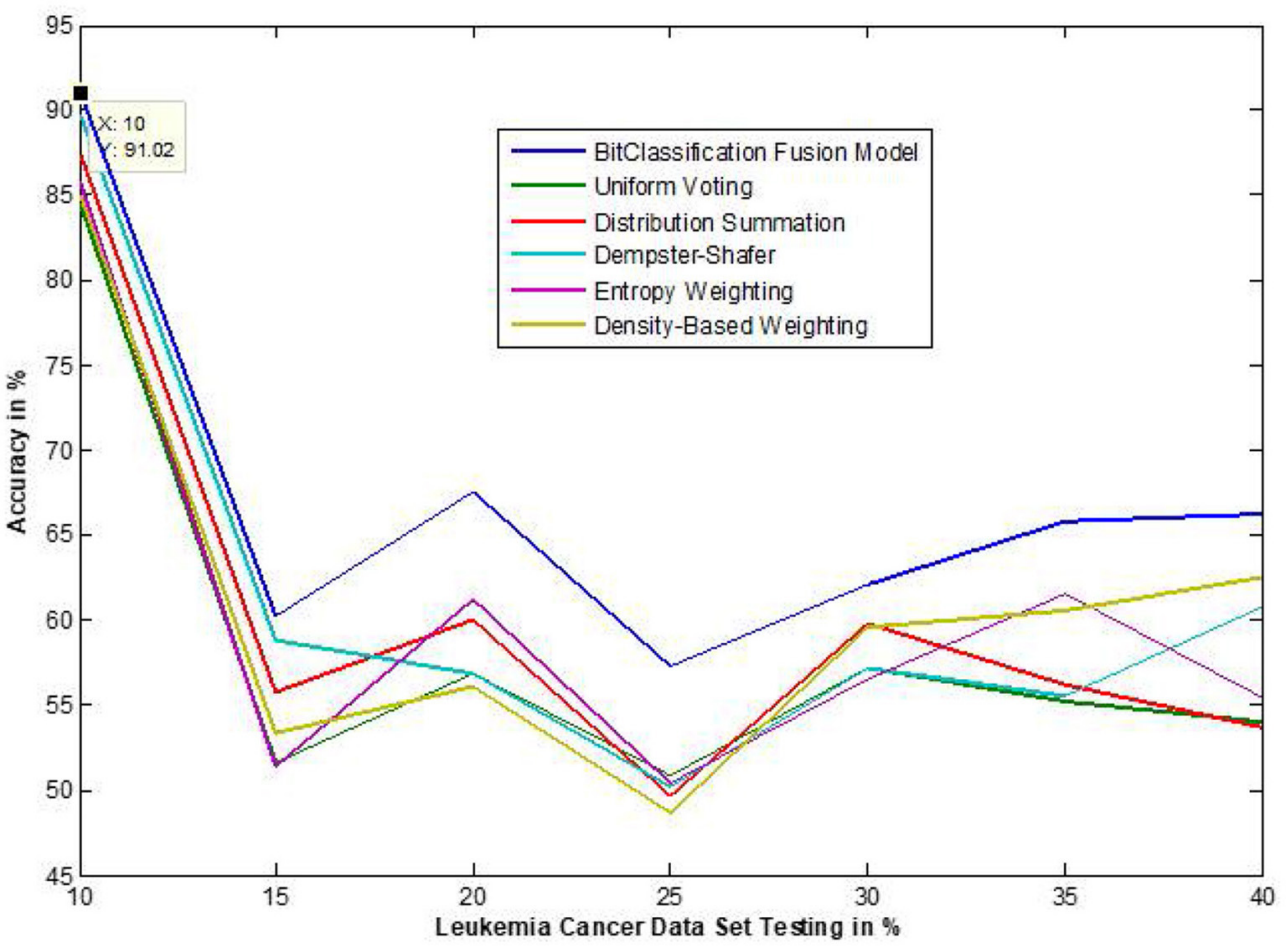
Accuracy performance bit fusion algorithm for Leukemia cancer with respect to other standard fusion techniques.

**Figure 10:**
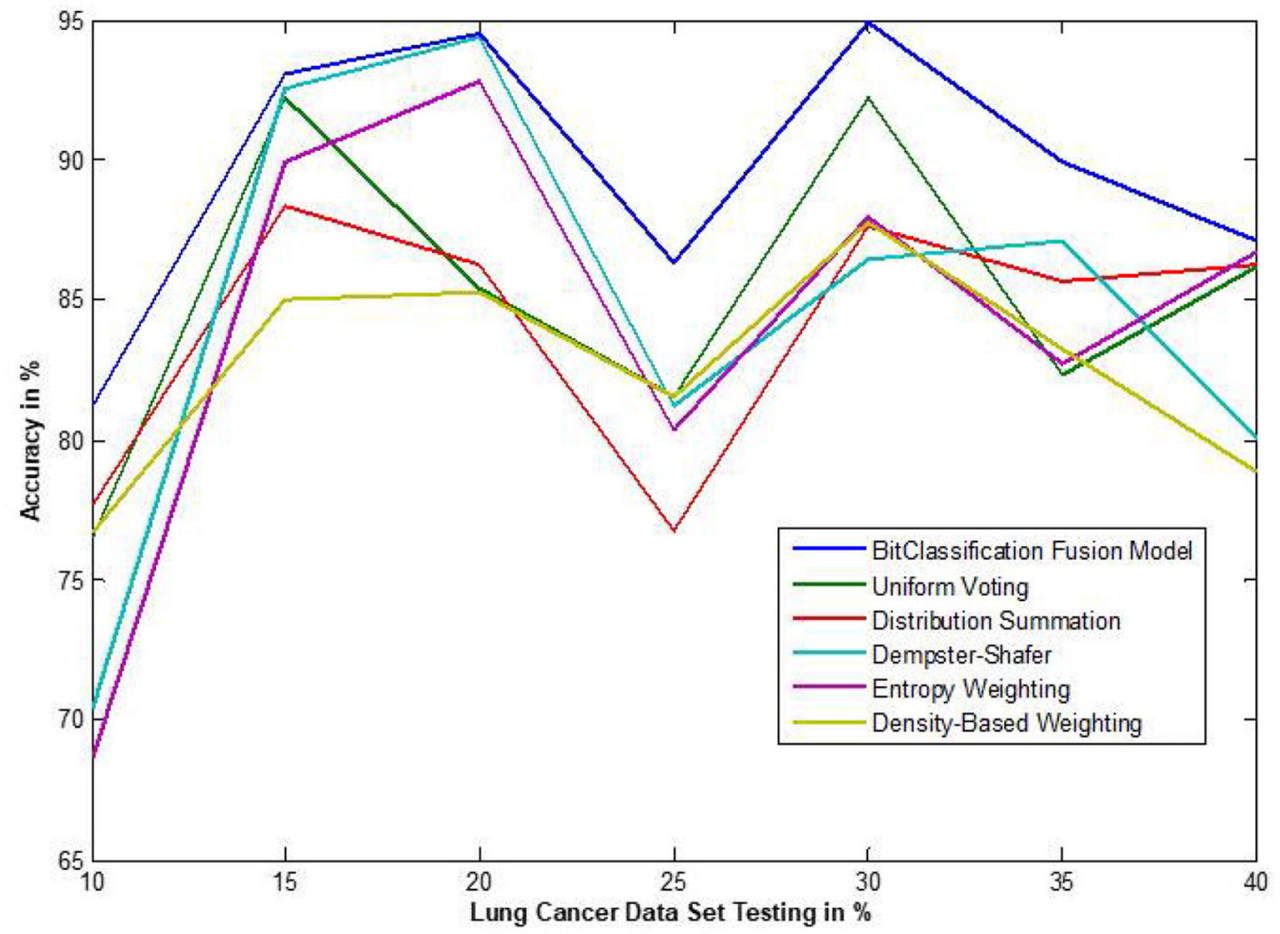
Accuracy performance bit fusion algorithm for Lung cancer with respect to other standard fusion techniques.

**Figure 11:**
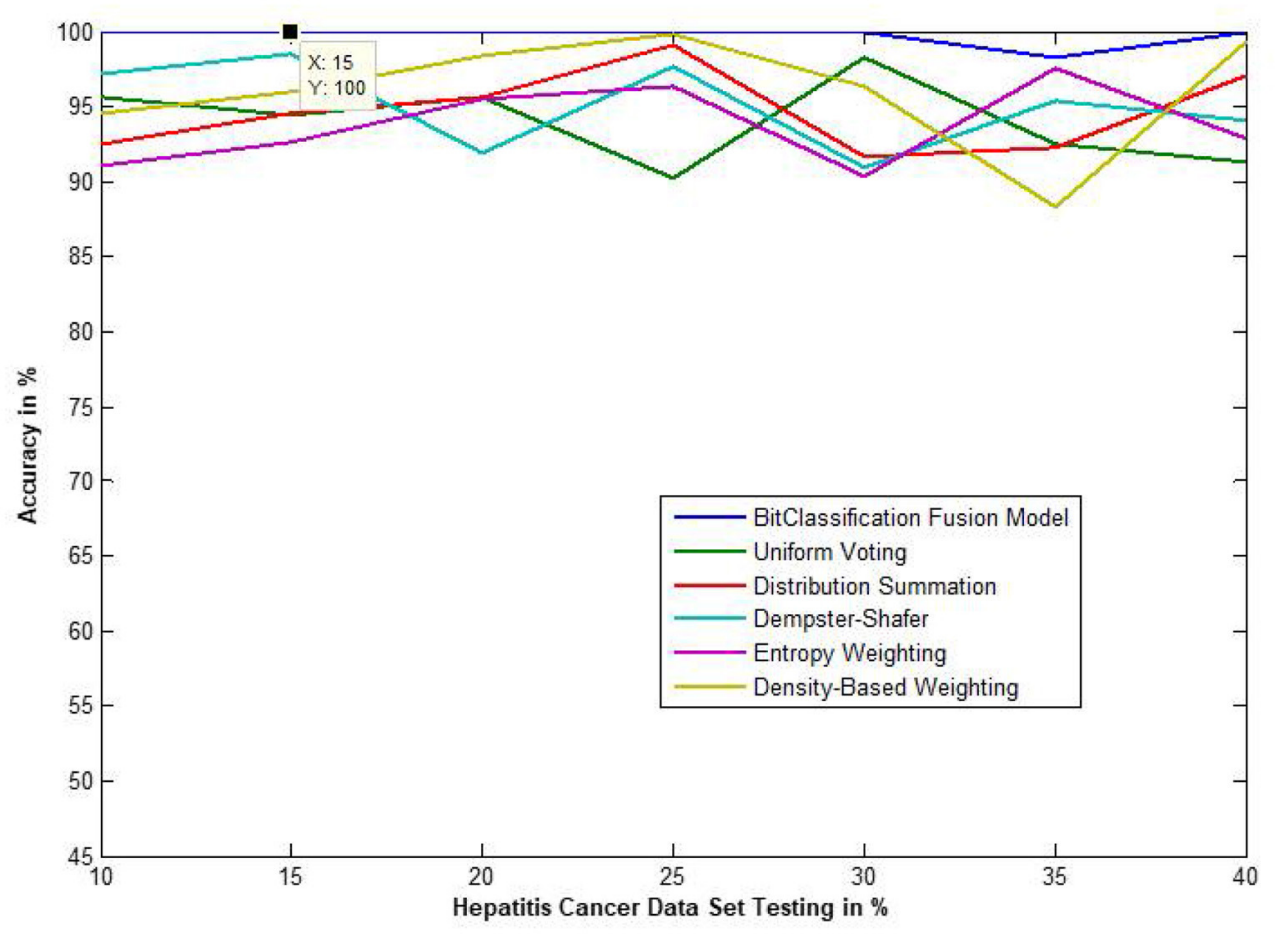
Accuracy performance of bit fusion algorithm for Hepatitis cancer with respect to other standard fusion techniques.

**Figure 12:**
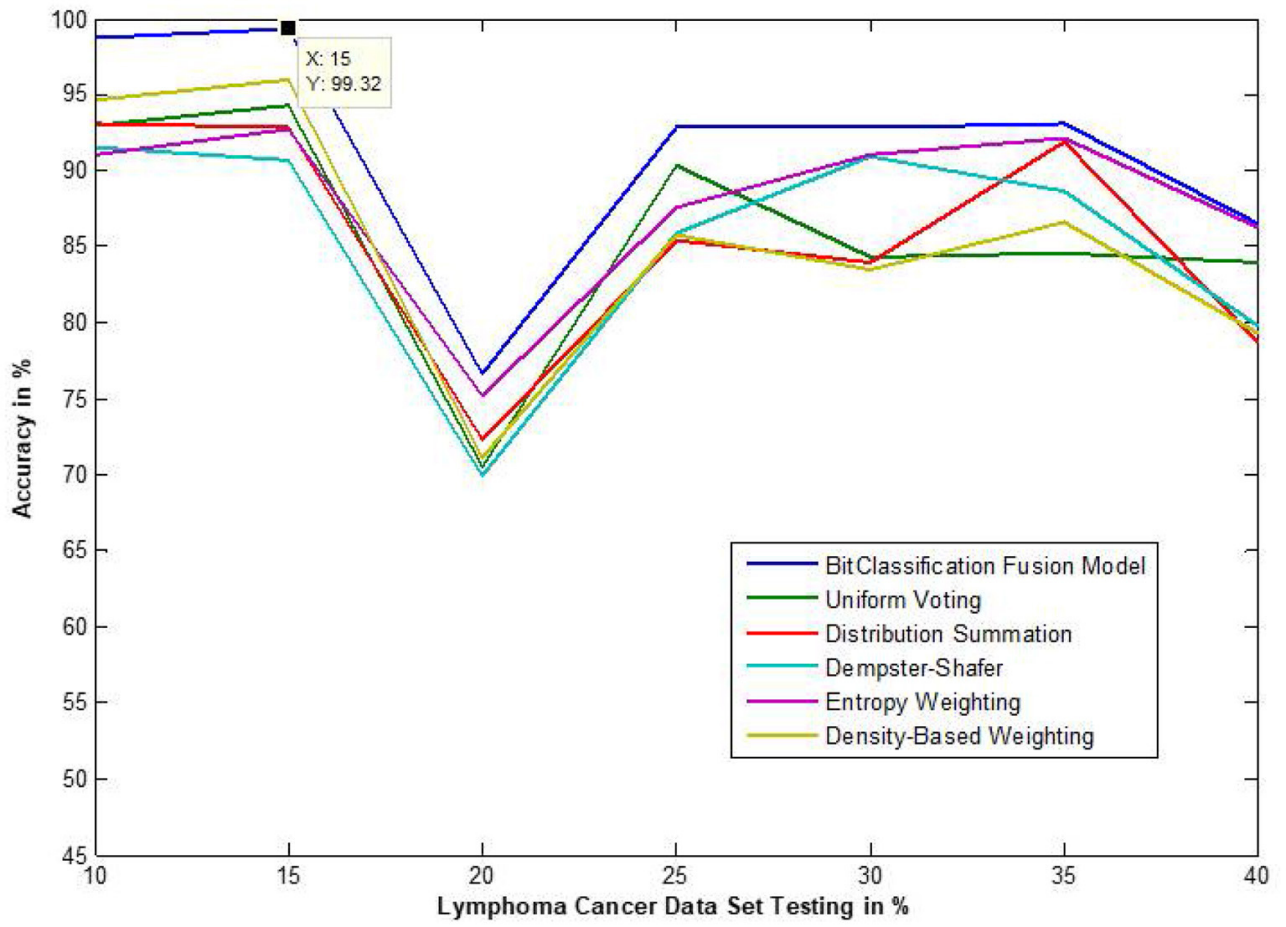
Accuracy performance of bit fusion algorithm for Lymphoma cancer with respect to other standard fusion techniques.

**Figure 13:**
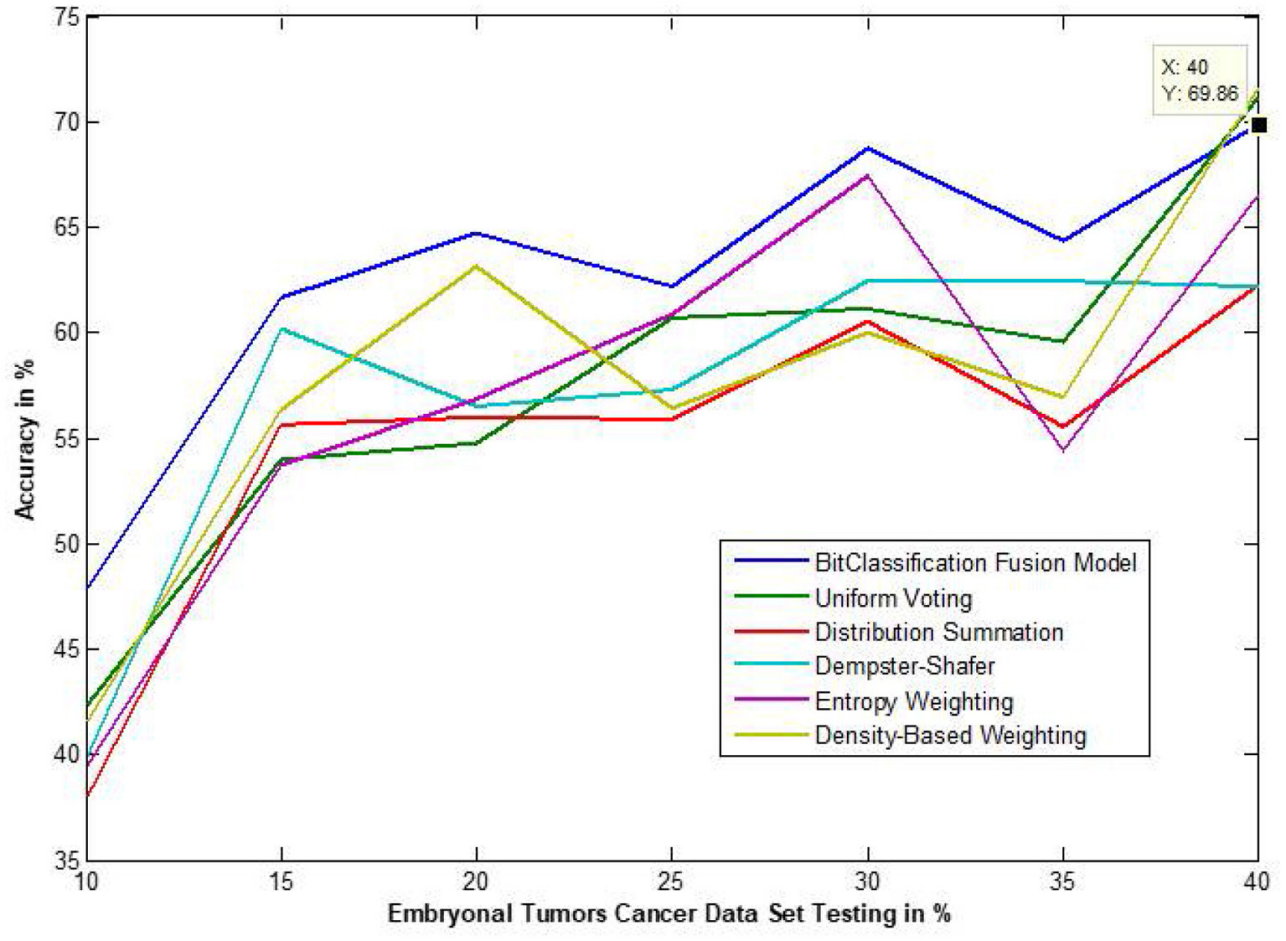
Accuracy performance of bit fusion algorithm for Embryonal Tumors cancer with respect to other standard fusion techniques.

The traditional fusion methods such asmajority voting, uniform distribution, distribution summation, Dempster-Shafer, Entropy Weighting and Density-based weighting take the individual input from each of the (1–6) base classifiers. As discussed in introduction, a number of fusion methods operate on the classifier's outputs trying to improve the classification accuracy. For example; in majority voting, if the greater number of classifiers predicts that, the instance belongs to the class 1 then automatically the fusion algorithm assigns class 1 as its class label to that instance. But in some cases; the accuracy may be decreased if the data belongs to some other class. In majority voting, the Time complexity would be high, but it increases the efficacy (13). The fusion methods play dominant role to enhance the accuracy of classification problem. Choosing the proper fusion method is one of the best solutions for any pattern recognition problem. Proposed bit-fusion ensemble classifier addresses the problem of tradition fusion methods, as it neither rely the number of classifiers nor the output of the base classifiers, it decides the output of a data element by tuning its own parameter and takes the decision according to the threshold δ value. We have implemented and compared our model with different traditional fusion methods as discussed above and the accuracy achieved by all the methods are shown in Figures 8–13 for different data sets, where x-axis represents % of data used for testing and y-axis represent accuracy. The 10-fold cross validation scheme has been implemented for training and testing of the data sets. Table 2 shows the average rate comparison with traditional fusion methods. Table 3, shows the similar comparison with DT and VWDT algorithms (49). The results show that the bit-fused ensemble classification just as effective as the other more complicated schemes in improving the recognition rate for the data set used.

**Table 3:** Performance on the UCI data sets (in %) with DT and VWDT (49).

**Dataset name**	**Average error rate**		
	**DT**	**VWDT**	**Proposed algorithm**
Glass	41.367	42.538	26.258
Bupa	24.751	20.526	12.265
Pima	26.042	23.828	13.241
Thyroid	14.484	3.32	2.210
Wine	6.825	2.857	1.105
German	27.564	24.154	11.023
Heart	17.407	15.926	9.205
Vehicle	33.394	26.195	18.125

**Figure 14:**
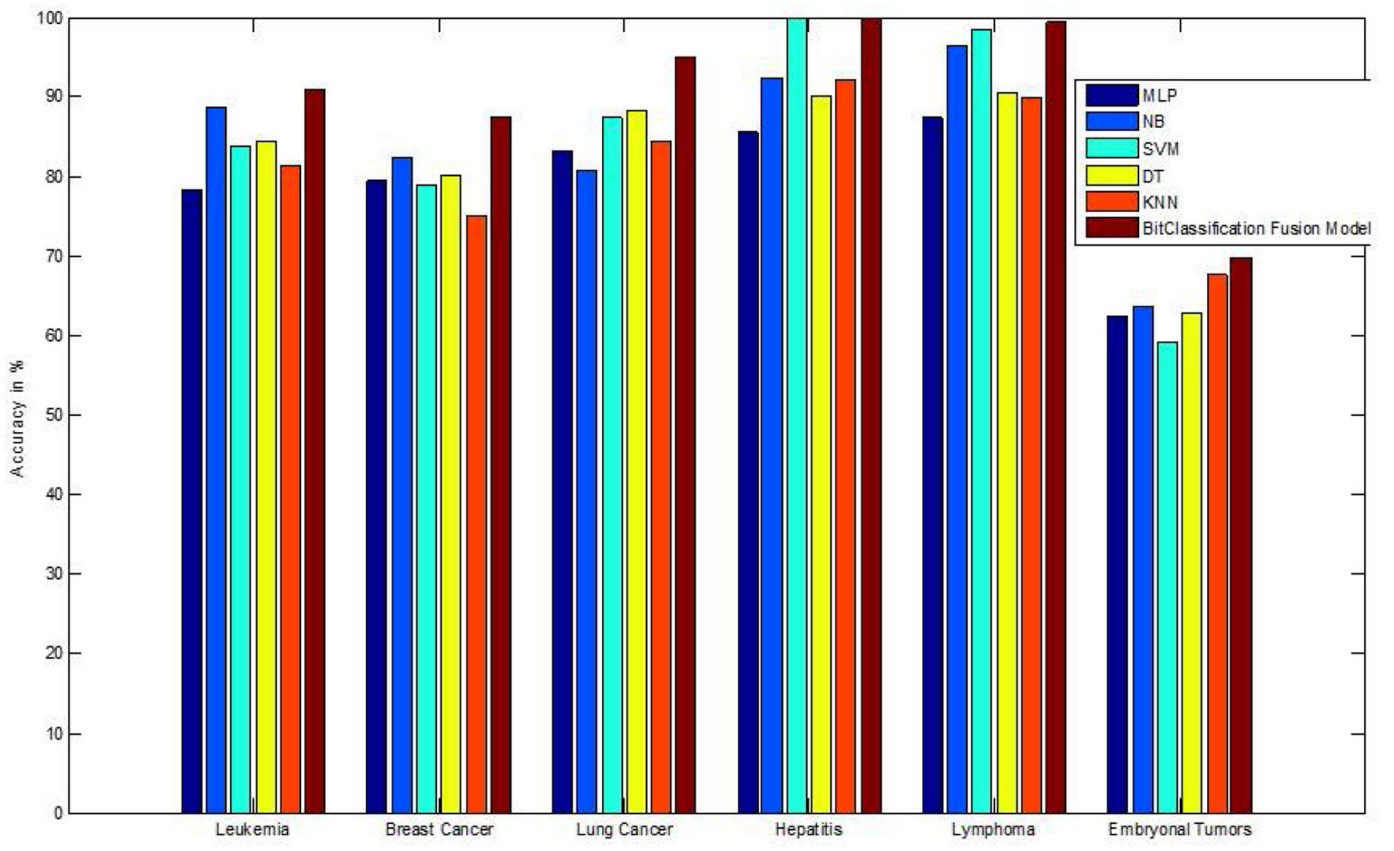
Comparative analysis of all dataset accuracies.

We have also measured the performance difference of individual classifiers with the proposed method. Figures 8–13 depicts the same. Our proposed algorithm performs 3–5% better accuracy than other algorithms in almost all cases. It can be noted from Figure 14 that SVM has good accuracy for dataset hepatitis and lymphoma, whereas N.B. is good with leukemia, KNN is better in the Embryonal dataset. But our proposed bit-fusion classifier outperforms all. This also proves our hypothesis that we cannot identify or rely on anyone classifier from the beginning, and it is better to fuse their result and classify one.

### Evaluation Comparison of Proposed Model With Logistic Regression and Fuzzy Integral

We have compared our algorithm with the findings from (50) on the 15 Benchmark data selected from the UEA & UCR Time Series Classification Repository (48), which details are given in Figure 7. To measure the accuracy obtained from the fusion classifier, we compared it with the best classifier result. Denoting n as the number of samples of the dataset and partitioned into k-fold with m number of pieces in each partition such that n = k ^*^ m, proposed model accuracy gain is measured using (17).


(17) $$\begin{array}{l} Acc_{j}=\;\frac{P_{j}-S_{j}}{m} \end{array}$$

Where, *Acc*_*j*_ is the accuracy gain on *the j*^th^ sample, *P*_*j*_ and *S*_*j*_ are the number of *j*^th^ samples correctly predicted by the proposed model and best classifier, respectively. For *k*, different result average gain for the dataset is evaluated using (18).


(18) $$\begin{array}{l} ACC\;=\;\sum_{j=1}^{k}\frac{Acc_{j}}{k} \end{array}$$

Accuracy is plotted in Figure 14, for the dataset discussed in Figure 7, to investigate and compare the impact of the proposed model with Majority Voting (MV), Highest Rank (H.R.), Borda Count (BC), Bayes Belief Integration (BBI), Behavior knowledge Space (BKS), Logistic Regression (L.R.) and Fuzzy Integral (F.I.).

**Figure 15:**
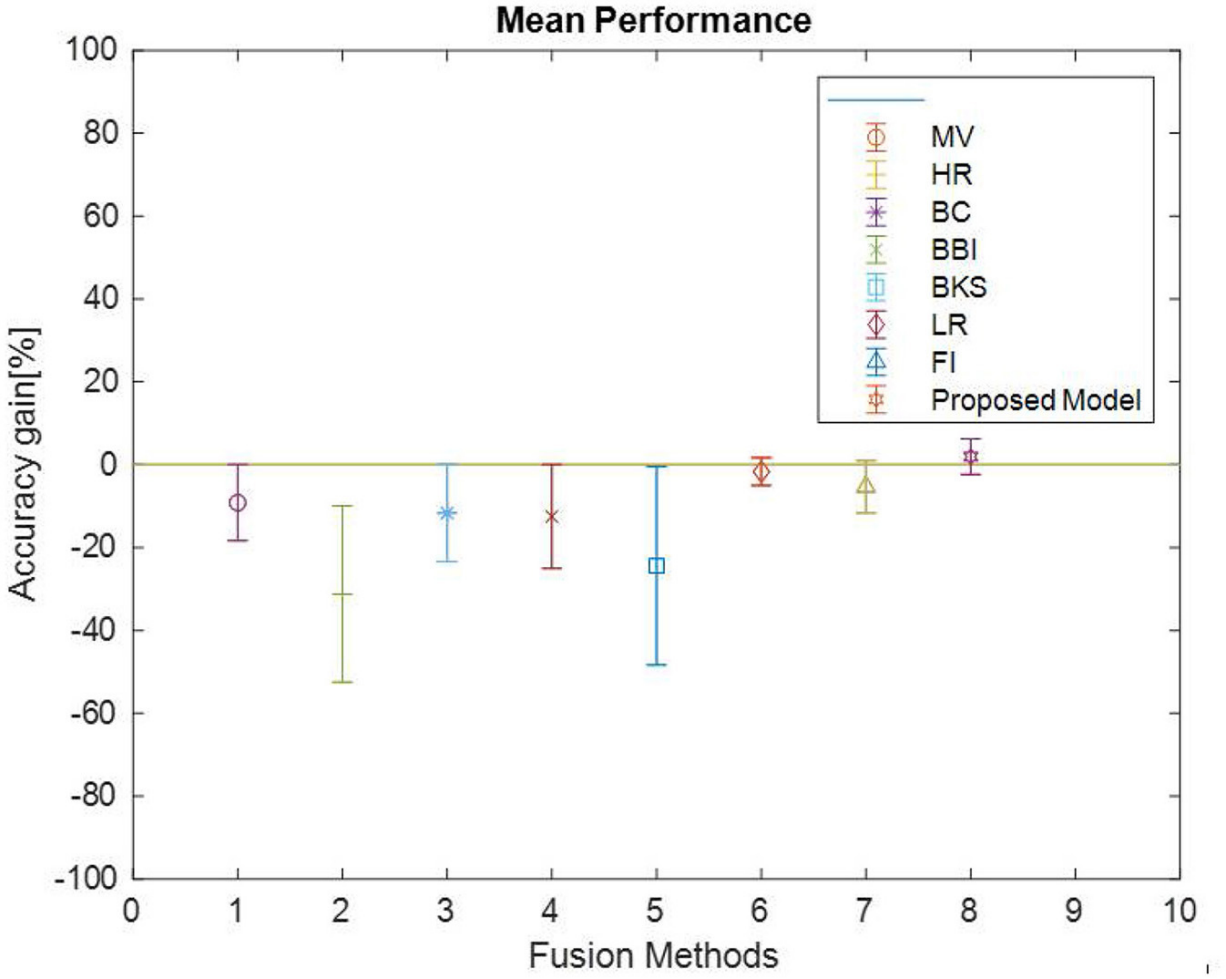
Fusion algorithm accuracy (mean, min, max) comparison.

It is observed from Figure 15 that the proposed model's mean accuracy in % is better than other methods. Considering the best scenario Proposed model has given the best performance in all 15 datasets. This can be confirmed from Table 4. However, the second-best is the L.R. method, but effective mean accuracy is still negative. The average accuracy % of BBI, MV, and BCI is in the range of −5 to −7%. In comparison, H.R. and BKS have performed worst for all the datasets.

**Table 4:** Comparative analysis of each fusion method with proposed model on different dataset for mean accuracy gain (48).

**Dataset**	**Majority voting**	**Highest rank**	**Borda count**	**Bayes belief integration**	**Behavior knowledge space**	**Logistic regression**	**Fuzzy Integral**	**Proposed model**
Beef	−18.33	−38.33	−23.33	−25	−48.33	−5	−11.67	−2.34
Chlorine concentration	−16.39	−52.52	−15.46	−0.02	−5.27	0	−0.14	3.4
Earthquakes	−2.59	−24.07	−1.73	0	−1.95	−0.43	−4.76	1.5
ECG5000	−0.66	−25.3	−0.76	−1.68	−2.78	0.08	−0.52	6.2
Face four	−1.78	−32.02	−2.69	−10.63	−26.68	−1.78	−2.69	1.5
FordA	−14.83	−24.36	−15.14	−2.48	−3.35	−0.06	−6.38	−0.04
Large kitchen appliances	−8.13	−22.27	−5.73	−4.8	−14.13	0.93	−2.4	4.5
Meat	0	−22.5	0	−7.5	−10	0	−5.83	0.5
Olive oil	−5	−10	−5	−11.67	−16.67	−3.33	−5	−1.4
OSU leaf	−15.63	−28.95	−12.89	−4.08	−35.28	0	−3.42	3.2
Symbols	−1.27	−12.06	−2.25	−3.24	−19.31	0.39	−0.78	2.1
Synthetic control	−0.17	−30.67	−1.33	0	−17.17	1.67	1	3.7
Trace	−9	−32	−13	−3.5	−19	−5	−2.5	−1.5
Two lead ECG	−1.38	−31.24	−1.63	−0.17	−0.52	−0.26	−0.09	3.3
Worms	−9.28	−20.94	−2.71	−5.81	−25.13	−1.52	−1.95	1.1

## Conclusion

This article focuses on the extensive implementation of fusion algorithms with a variety of datasets, proposed model deals with a novel bit fusion algorithm that contemplates the input as soft class label applied on gene expression standard datasets. The proposed Bit-fused ensemble algorithm is an active and reasonably robust fusion structure that outpaces the standard and many other present fusion approaches compared to accuracy, time complexity and correctness. The proposed Bit-fusion compares the data feature-wise with the threshold value and classifies each feature as the soft class label. The algorithm focuses on diversity measurement as compared to other existing methodologies. After the classification result, traditional algorithms are combined to enhance accuracy. Figure 15 and Table 4 reflect accuracy gain compared for seven traditional fusion algorithms with the datasets.

The proposed methodology rises the correctness by focusing on categorizing each value of the feature rather than categorizing the whole feature itself. High accuracy for the large data set with little additional computational determination can be accomplished with the model. Future work may concentrate on the pandemic data (Covid data) to classify with a novel bit fusion algorithm and predict the type of patients or the cluster area by using the proposed bit-fused algorithm as it works on individual features value it can establish the higher correctness of the result. A variety of other base classifiers may be used to establish the correctness of the proposed algorithm with a variety of datasets. Various optimization techniques maybe used in bit- fusion to enhance the accuracy and to deal with large datasets.

## Data Availability Statement

Publicly available datasets were analyzed in this study. This data can be found at: UCI, UEA, and UCR repositories.

## Author Contributions

SM, DM, and KS: methodology, visualization, and writing—original draft. KS: conceptualization. SM, DM, SP, KK, SK, and SB: writing—review and editing. SM and DM: data curation and software. SM, DM, KS, SK, and SB: investigation. SK and SM: super-vision. All authors contributed to the article and approved the submitted version.

## Funding

This work has received limited APC support from Symbiosis International Deemed University.

## Conflict of Interest

The authors declare that the research was conducted in the absence of any commercial or financial relationships that could be construed as a potential conflict of interest.

## Publisher's Note

All claims expressed in this article are solely those of the authors and do not necessarily represent those of their affiliated organizations, or those of the publisher, the editors and the reviewers. Any product that may be evaluated in this article, or claim that may be made by its manufacturer, is not guaranteed or endorsed by the publisher.

## References

[1] Xu L Krzyzak A Suen C. Methods of combining multiple classifiers and their applications to hand written numerals. IEEE Trans. Syst Man Cybern. (1992) 22:418–35. 10.1109/21.155943

[2] Hanczar B Bar-Hen A. A new measure of classifier performance for gene expression data. IEEE Trans Comput Biol Bioinform. (2012) 95:1379–86. 10.1109/TCBB.2012.21 22291161

[3] Kilic E Alpaydin E. Learning the areas of expertise of classifiers in an ensemble. Proc. Computer Science. (2011) 3:74–82. 10.1016/j.procs.2010.12.014

[4] Hazem JM Bakry E. An efficient algorithm for pattern detection using combined classifiers and data fusion. Inf Fusion. (2010) 11:133–48. 10.1016/j.inffus.2009.06.001

[5] Hassanien AE Milanova MG Smolinski TG Abraham A. Computational intelligence in solving bioinformatics problems: Reviews, perspectives, and challenges. In: Computational Intelligence in Biomedicine and Bioinformatics. Berlin: Springer (2008). p. 3–47.

[6] Kittler J. On combining classifiers. IEEE Trans Pattern Anal Mach Intell. (1998) 20:226–39. 10.1109/34.667881

[7] JAIN AK Duin RPW Jianchang M. Statistical pattern recognition: a review. IEEE Trans Pattern Anal Mach Intell. (2000) 22:4–37. 10.1109/34.824819

[8] Enriquez F Cruz FL Javier Ortega F Vallego CG Troyano JA. A comparative study of combination applied to NLP tasks. Inf Fusion. (2013) 14:255–67. 10.1016/j.inffus.2012.05.001

[9] Shah C Jivani AG. Comparison of data mining classification algorithms for breast cancer prediction. In: 2013 Fourth International Conference on Computing, Communications and Networking Technologies. IEEE (2013). p. 1–4.

[10] Opitz D Maclin R. Popular ensemble methods: an empirical study. J Artif Intell Res. (1999) 11:169–98. 10.1613/jair.614

[11] Bagheri MA Gao Q Escalera S. Logo recognition based on the dempster-shafer fusion of multiple classifiers. In: Canadian Conference on Artificial Intelligence. Berlin: Springer (2013). p. 1–12.

[12] Sohn SY Lee SH. Data fusion, ensemble and clustering to improve the classification accuracy for the severity of road traffic accidents in korea. Safety Science. (2003) 41:1–14. 10.1016/S0925-7535(01)00032-7

[13] Saxena U Moulik S Nayak SR Hanne T Roy DS. “Ensemble-based machine learning for predicting sudden human fall using health data,” in Mathematical Problems in Engineering. (2021). p. 1–12.

[14] Namamula LR Chaytor D. Effective ensemble learning approach for large-scale medical data analytics. Int J Syst Assur Eng Manag. (2022) 1–8. 10.1007/s13198-021-01552-7

[15] Vo K Jonnagaddala J Liaw ST. Statistical supervised meta-ensemble algorithm for medical record linkage. J Biomed Inform. (2019) 95:103220. 10.1016/j.jbi.2019.103220 31158554

[16] Nagarajan SM Muthukumaran V Murugesan R Joseph RB Munirathanam M. Feature selection model for healthcare analysis and classification using classifier ensemble technique. Int J Syst Assur Eng Manag. (2021). 10.1007/s13198-021-01126-7

[17] Ihnaini B Khan MA Khan TA Abbas S Daoud MS Ahmad M. A smart healthcare recommendation system for multidisciplinary diabetes patients with data fusion based on deep ensemble learning. Comput Intell Neurosci. (2021) 2021:4243700. 10.1155/2021/4243700 34567101 PMC8463188

[18] Abdelhalim A Traore I. A new method for learning decision trees from rules. In: 2009 International Conference on Machine Learning and Applications. IEEE (2009). p. 693–8.

[19] Quinlan JR. Introduction of decision trees. Mach Learn. (1986) 1:81–106. 10.1007/BF00116251

[20] Patil DV Bichkar RS. Issues in optimization of decision tree learning: A survey. Int J Appl Infm Syst. (2012) 3:1–18. Available online at: https://citeseerx.ist.psu.edu/viewdoc/download?doi=10.1.1.401.9418&rep=rep1&type=pdf

[21] Goin JE. Classification bias of the k-nearest neighbor algorithm. IEEE Trans. Pattern Anal Mach Intell. (1984) 6:379–81. 10.1109/TPAMI.1984.4767533 21869207

[22] Eric B Baum. On the capabilities of multilayer perceptrons. J Complex. (1988) 4:193–215. 10.1016/0885-064X(88)90020-9

[23] Devasena CL. Efficiency comparison of multilayer perceptron and smo classifier for credit risk prediction. Int J Adv Res Comput Commun Eng. (2014) 3:6156–62. Available online at: https://ijarcce.com/wp-content/uploads/2012/03/IJARCCE1B-a-lakshmi-devasena-Efficiency-Comparison-of-Multilayer.pdf

[24] Sibanda W Pretorius P. Novel application of multi-layer perceptrons (MLP) neural networks to model HIV in South Africa using seroprevalence data from antenatal clinics. Int J Comput Appl. (2011) 35:26–31. 10.5120/4398-6106

[25] Shankar K Pal Mitra S. Multi layer perceptron fuzzy sets and classification. IEEE Trans Neural Netw. (1992) 3:683–97. 10.1109/72.159058 18276468

[26] Suykens JA Vandewalle J. Training multilayer perceptron classifiers based on a modified support vector method. IEEE Trans Neural Netw. (1999) 10:907–11. 18252586 10.1109/72.774254

[27] Helman P Veroff R Atlas SR Willman C. A bayesian network classification methodology for gene expression data. J Comput Biol. (2004) 11:581–615. 10.1089/cmb.2004.11.581 15579233

[28] Cheng J Greiner R. Comparing bayesian network classifiers. In: UAI'99: Proceedings of the Fifteenth Conference on Uncertainty in Artificial Intelligence. (1999). p. 101–8.

[29] Chickering DM Heckerman D. Efficient approximations for the marginal likelihood of Bayesian networks with hidden variables. Machine Learn. (1997) 29:181–212.

[30] Tong M Hong Liu K Xu C Ju W. An ensemble of svm classifiers based on gene pairs. Comput Biol Med. (2013) 43:729–37. 10.1016/j.compbiomed.2013.03.010 23668348

[31] Thadani K Jayaraman VK Sundararajan V. Evolutionary selection of kernels in support vector machines. In: 2006 International Conference on Advanced Computing and Communications. IEEE (2006). p. 19–24.

[32] Chen Z Li J Wei L Xu W Shi Y. Multiple-kernel SVM based multiple-task oriented data mining system for gene expression data analysis. Expert Syst Appl. (2011) 38:12151–9. 10.1016/j.eswa.2011.03.025

[33] Cortes C Vapnik V. Support vector networks. Mach Learn. (1995) 20:273–97. 10.1007/BF00994018

[34] Colin C Nello C. Simple Learning Algorithms for Training Support Vector Machines. Technical Report, University of Bristol, Bristol, United Kingdom (1998).

[35] Tsiliki G Kossida S. Fusion methodologies for biomedical data. J Proteomics. (2011) 74:2774–85. 10.1016/j.jprot.2011.07.001 21767675

[36] Reboiro Jato M Diaz F Glez-Pena D Fdez-Riverola F. A novel ensemble of classifiers that use biological relevant gene sets for micro-array classification. Appl Soft Comput. (2014) 17:117–26. 10.1016/j.asoc.2014.01.002

[37] Morrison D De Silva LC. Voting assembles of spoken affect classification. J Netw Comput Appl. (2007) 30:1356. 10.1016/j.jnca.2006.09.005

[38] Ludmila Kuncheva I Lakhmi Jain C. Designing classifier fusion systems by genetic algorithms. IEEE Trans Evol Comput. (2000) 4:327–36. 10.1109/4235.887233

[39] Ludmila Kuncheva I. A theoretical study on six classifier fusion strategies. IEEE Trans Pattern Anal Mach Intell. (2002) 24:281–6. 10.1109/34.982906

[40] Ramos Terrades O Valveny E Tabbone S. Optimal classifier fusion in a non-bayesian probabilistic framework. IEEE Trans Pattern Anal Mach Intell. (2009). 31:1630–44. 10.1109/TPAMI.2008.224 19574623

[41] Golub TR Slonim DK Tamayo P Huard C Gaasenbeek M Mesirov JP. Molecular classification of cancer: class discovery and class prediction by gene expression monitoring. Science. (1999) 286:531–7. 10.1126/science.286.5439.531 10521349

[42] Available online at: http://archive.ics.uci.edu/ml/datasets/ Breast+ Cancer+Wisconsin + (Diagnostic) (1988).

[43] Hong Z Yang JY. Optimal discriminant plane for a small number of samples and design method of classifier on the plane. Pattern Recogn. (1991) 24:317–24. 10.1016/0031-3203(91)90074-F

[44] http://archive.ics.uci.edu/ml/datasets/Hepatitis (accessed January 12, 2022) (1988).

[45] Alizadeh AA Eisen MB Davis RE Ma C Lossos IS Rosenwald A. Distinct types of diffuse large B-cell lymphoma identified by gene expression profiling. Nature. (2000) 403:503–11. 10.1038/35000501 10676951

[46] Pomeroy SL Tamayo P Gaasenbeek M Sturla LM Angelo M McLaughlin ME. Gene expression-based classification and outcome prediction of central nervous system embryonal tumors. Nature. (2002) 415:436–42. 10.1038/415436a 11807556

[47] Shi L Campbell G Jones WD Campagne F Wen Z Walker SJ. The MicroArray Quality Control (MAQC)-II study of common practices for the development and validation of microarray-based predictive models. Nat Biotechnol. (2010) 28:827. 10.1038/nbt.1665 20676074 PMC3315840

[48] Bagnall A Lines J Bostrom A Large J Keogh E. The great time series classification bake off: a review and experimental evaluation of recent algorithmic advances. Data Min Knowl Discov. (2017) 31:606–60. 10.1007/s10618-016-0483-9 30930678 PMC6404674

[49] Aizhong M Lei W Junyan Q. A multiple classifier fusion algorithm using weighted decision templates. Scientific Program. (2016) 10:3943859. 10.1155/2016/3943859

[50] Söffker D Kudszus B Rothe S. Does classifier fusion improve the overall performance numerical analysis of data and fusion method characteristics in?uencing classifier fusion performance. Entropy. (2019) 21:866. 10.3390/e21090866

